# Supplementary material for: Data‐Driven Discovery of Unconventional Antiferromagnets
Source: Adv Sci (Weinh). 2026 Jul 29:e76844. Online ahead of print. doi: 10.1002/advs.76844 (PMC13418068; doi:10.1002/advs.76844)
Supplement: Supplementary file 1 — Supporting File: advs76844‐sup‐0001‐SuppMat.pdf. [file ADVS-9999-e76844-s001.pdf]

Supporting Information for "Data-Driven Discovery of Unconventional Antiferromagnets"

Qirui Cui[1,2†], Chenxu Liu[3†], Anna Delin[1,2,5*], Kaiyou Wang[4,6*]

[1]Department of Applied Physics, School of Engineering Sciences, KTH Royal Institute of Technology, AlbaNova University Center, SE-10691 Stockholm, Sweden.
[2]Swedish e-Science Research Center, KTH Royal Institute of Technology, SE-10044 Stockholm, Sweden.
[3]Faculty of Science and Engineering, University of Nottingham Ningbo China, Ningbo 315100, China.
[4]State Key Laboratory of Superlattices and Microstructures, Institute of Semiconductors, Chinese Academy of Sciences, Beijing 100083, China.
[5]Wallenberg Initiative Materials Science for Sustainability (WISE), KTH Royal Institute of Technology, SE-10044 Stockholm, Sweden.
[6]College of Materials Science and Opto-Electronic Technology, University of Chinese Academy of Sciences, Beijing, China.

*Corresponding author(s). E-mail(s): annadel@kth.se; kywang@semi.ac.cn;
†These authors contributed equally to this work.

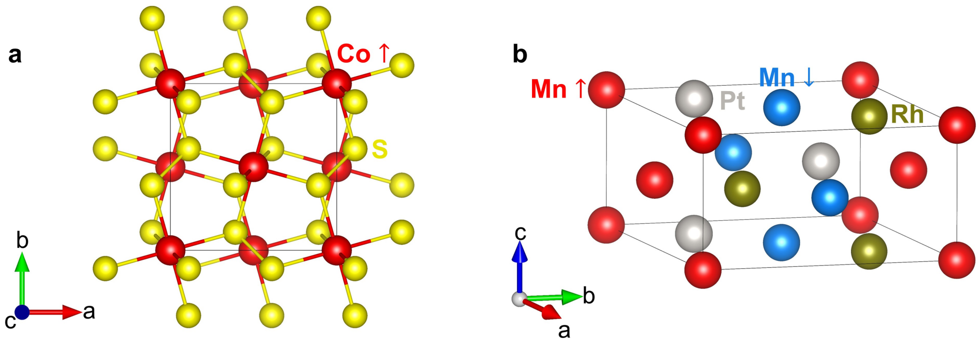

Figure S1. The crystal structure and magnetic structure of (a) Cos_2 and (b) Mn_2PtRh. The Mn atoms on the spin-up and spin-down sublattices are shown by red and blue spheres, respectively.

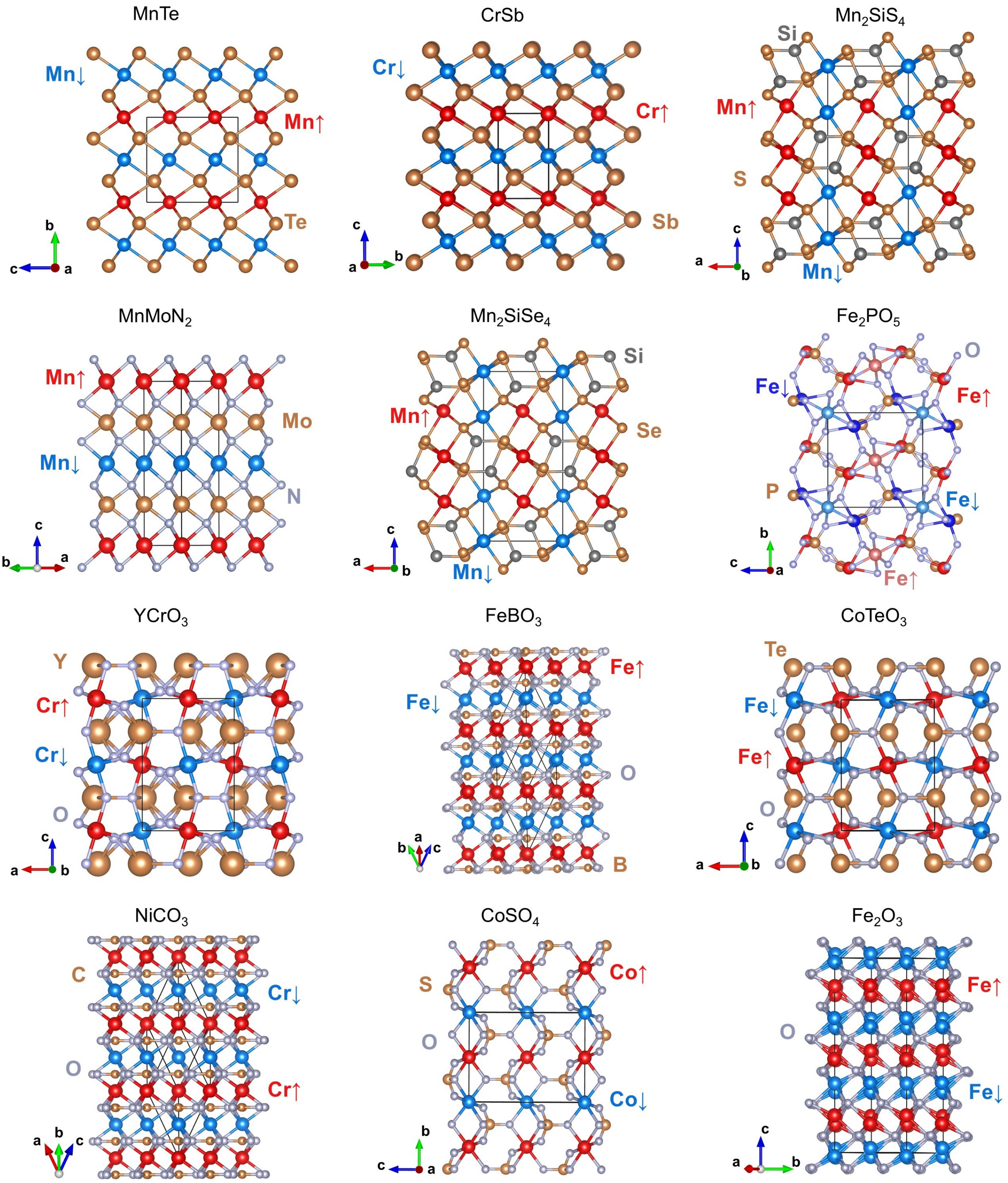

Figure S2. The crystal structures and magnetic structures of altermagnets MnTe, CrSb, Mn_2SiS_4, $MnMoN_2$, Mn_2SiSe_4, Fe_2PO5, $YCrO_3$, $FeBO_3$, $CoTeO_3$, $Nico_3$, $CoSO_4$, Fe_2O_3. The magnetic atoms on the spin-up and spin-down sublattices are shown by red and blue spheres, respectively.

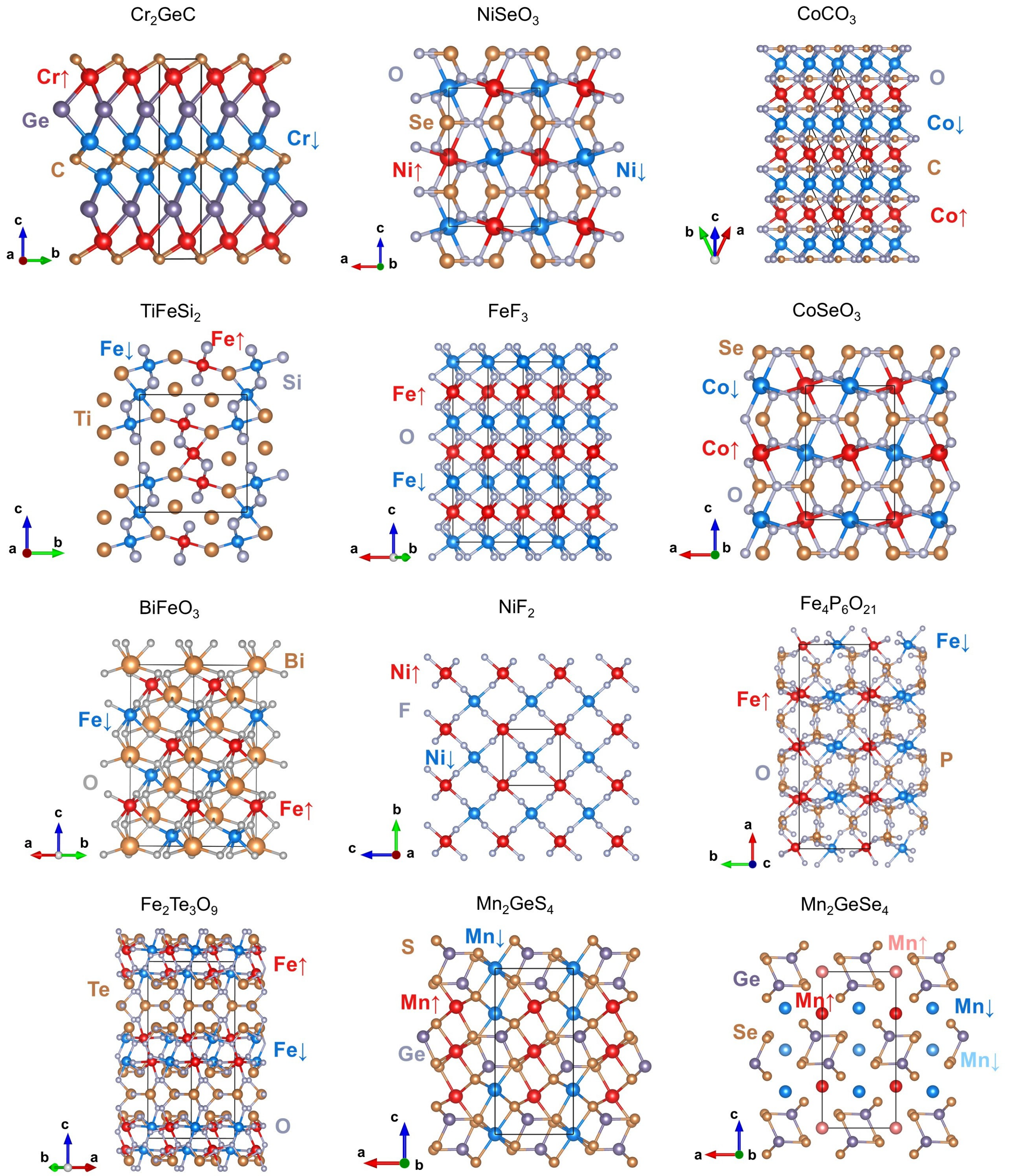

Figure S3. The crystal structures and magnetic structures of altermagnets Cr_2GeC, $NiSeO_3$, $CoCO_3$, $TiSeSi_2$, FeF_3, $CoSeO_3$, $BiFeO_3$, NiF_2, $Fe_4P_6O_2$1, $Fe_2Te_3O_9$, Mn_2GeS_4, Mn_2GeSe_4. The magnetic atoms on the spin-up and spin-down sublattices are shown by red and blue spheres, respectively.

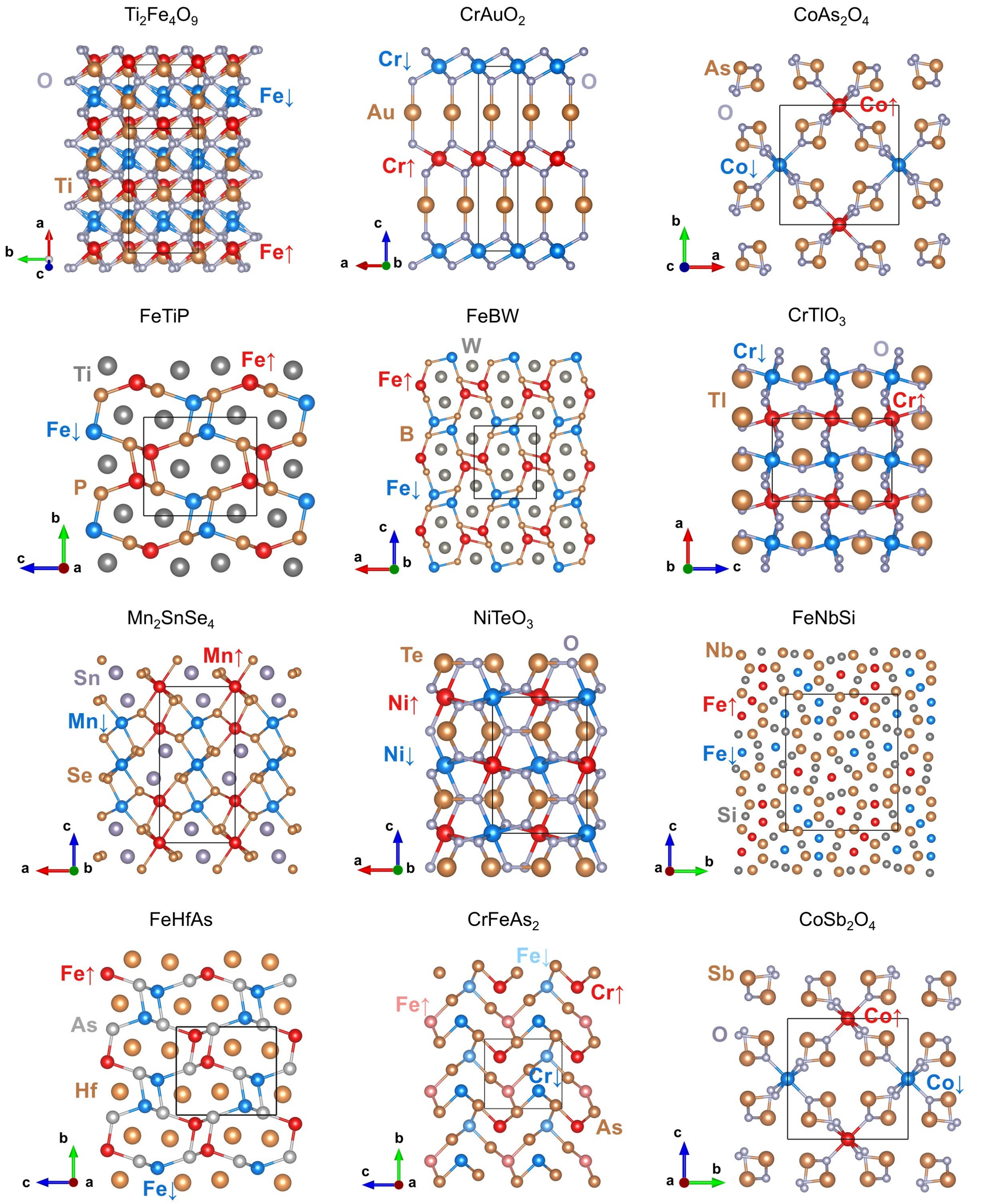

Figure S4. The crystal structures and magnetic structures of altermagnets $Ti_2Fe_4O_9$, $CrAuO_2$, $CoAs_2O_4$, FeTiP, FeBw, $CrTlO_3$, Mn_2SnSe_4, $NiTeO_3$, FeNbSi, HfFeAs, $CrFeAs_2$, $CoSb_2O_4$. The magnetic atoms on the spin-up and spin-down sublattices are shown by red and blue spheres, respectively.

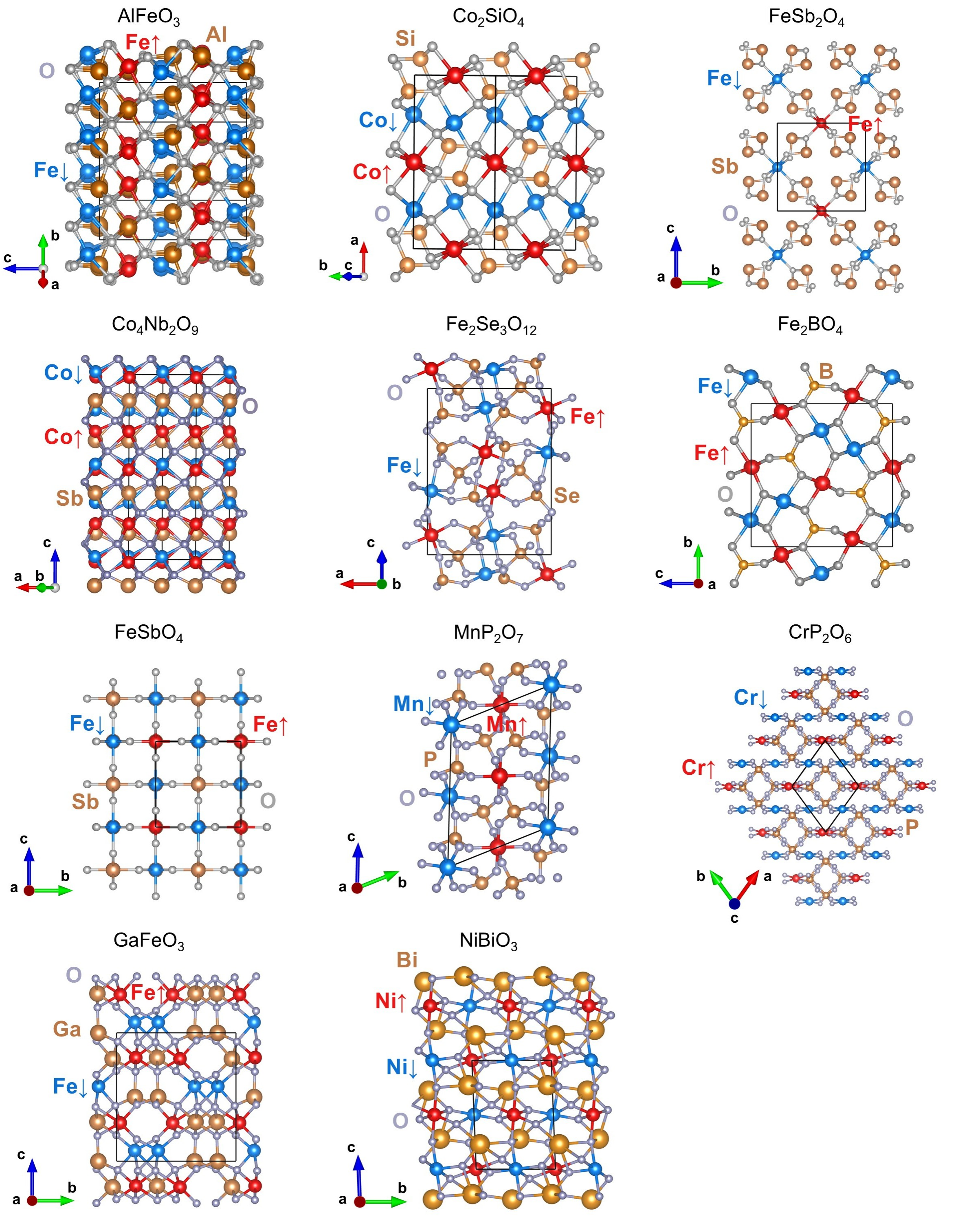

Figure S5. The crystal structures and magnetic structures of Luttinger-compensated ferrimagnets $AlFeO_3$, Co_2SiO_4, $FeSb_2O_4$, $Co_4Nb_2O_9$, $Fe_2Se_3O_{1}2$, Fe_2BO_4, $FeSbO_4$, MnP_2O_7, CrP_2O_6, $GaFeO_3$, $NiBiO_3$. The magnetic atoms on the spin-up and spin-down sublattices are shown by red and blue spheres, respectively.

Electronic band structures—For the reproduction of our electronic band structures, we list the high-symmetry points in reciprocal space for all 36 altermagnets and 11 Luttinger-compensated ferrimagnets studied in this work. The Γ point $(0,0,0)$ is identical for all candidates. 1. **Altermagnets**, MnTe: L_1 $(1/2,0,1/2)$, L_2 $(0,1/2,1/2)$; CrSb: L_1 $(1/2,0,1/2)$, L_2 $(0,1/2,1/2)$; Mn_2SiS_4: R_1 $(1/2,1/2,1/2)$, R_2 $(-1/2,1/2,1/2)$, L_1 $(1/2,0,1/2)$, L_2 $(0,1/2,1/2)$; Mn_2SiSe_4: U_1 $(1/2,0,1/2)$, U_2 $(-1/2,0,1/2)$; Fe_2PO_5: T_1 $(0,1/2,1/2)$, T_2 $(0,-1/2,1/2)$; $YCrO_3$: S_1 $(1/2,1/2,0)$, S_2 $(-1/2,1/2,0)$; $FeBO_3$: B_1 $(0.78,0.22,1/2)$, B_2 $(0.22,0.78.1/2)$; $CoTeO_3$: U_1 $(1/2,0,1/2)$, U_2 $(-1/2,0,1/2)$; $NiCO_3$: B_1 $(0.78,0.22,1/2)$, B_2 $(0.22,0.78.1/2)$; $CoSO_4$: T_1 $(0,1/2,1/2)$, T_2 $(0,-1/2,1/2)$; Fe_2O_3: H_1 $(1/3,1/3,1/2)$, H_2 $(-1/3,-1/3,1/2)$; Cr_2GeC: L_1 $(1/2,0,1/2)$, L_2 $(0,1/2,1/2)$; $NiSeO_3$: U_1 $(1/2,0,1/2)$, U_2 $(-1/2,0,1/2)$; $CoCO_3$: B_1 $(0.78,0.22,1/2)$, B_2 $(0.22,0.78.1/2)$; $TiFeSi_2$: T_1 $(0,1/2,1/2)$, T_2 $(0,-1/2,1/2)$; FeF_3: H_1 $(1/3,1/3,1/2)$, H_2 $(-1/3,-1/3,1/2)$; $CoSeO_3$: U_1 $(1/2,1/2,0)$, U_2 $(-1/2,1/2,0)$; $BiFeO_3$: H_1 $(1/3,1/3,1/2)$, H_2 $(-1/3,2/3,1/2)$; NiF_2: T_1 $(0,1/2,1/2)$, T_2 $(0,-1/2,1/2)$; $Fe_4P_6O_{21}$: A_1 $(1/2,1/2,0)$, A_2 $(-1/2,1/2,0)$; $Fe_2Te_3O_9$: U_1 $(1/2,0,1/2)$, U_2 $(-1/2,0,1/2)$; Mn_2GeS_4: U_1 $(1/2,0,1/2)$, U_2 $(-1/2,0,1/2)$; Mn_2GeSe_4: U_1 $(1/2,0,1/2)$, U_2 $(-1/2,0,1/2)$; $Ti_2Fe_4O_9$: R_1 $(1/2,1/2,1/2)$, R_2 $(-1/2,1/2,-1/2)$; $CrAuO_2$: L_1 $(1/2,0,1/2)$, L_2 $(0,1/2,1/2)$; $CoAs_2O_4$: S_1 $(1/2,1/2,0)$, S_2 $(-1/2,1/2,0)$; FeTiP: T_1 $(0,1/2,1/2)$, T_2 $(0,-1/2,1/2)$; FeBW: U_1 $(1/2,0,1/2)$, U_2 $(-1/2,0,1/2)$; $CrTlO_3$: S_1 $(1/2,1/2,0)$, S_2 $(-1/2,1/2,0)$; Mn_2SnSe_4: U_1 $(1/2,0,1/2)$, U_2 $(-1/2,0,1/2)$; $NiTeO_3$: U_1 $(1/2,0,1/2)$, U_2 $(-1/2,0,1/2)$; FeNbSi: T_1 $(0,1/2,1/2)$, T_2 $(0,-1/2,1/2)$; FeHfAs: T_1 $(0,1/2,1/2)$, T_2 $(0,-1/2,1/2)$; $CrFeAs_2$: S_1 $(0,-1/2,1/2)$, S_2 $(0,1/2,1/2)$; $CoSb_2O_4$: T_1 $(0,1/2,1/2)$, T_2 $(0,-1/2,1/2)$. 2. **Luttinger-compensated ferrimagnets**, $AlFeO_3$: X $(1/2,0,0)$, S $(1/2,1/2,0)$, Y $(0,1/2,0)$, Z $(0,0,1/2)$, U $(1/2,0,1/2)$, R $(1/2,1/2,1/2)$, T $(0,1/2,1/2)$; Co_2SiO_4: B $(0,1/2,0)$, D $(0,1/2,1/2)$, X $(0,0,1/2)$, Z $(1/2,0,0)$; $FeSb_2O_4$: X $(1/2,0,0)$, Y $(0,1/2,0)$, Z $(0,0,1/2)$, R $(1/2,1/2,1/2)$, T $(0,1/2,1/2)$, U $(1/2,0,1/2)$; $Co_4Nb_2O_9$: X $(1/2,0,0)$, Y $(0,1/2,0)$, Z $(0,0,1/2)$, R $(-1/2,-1/2,1/2)$, L $(2/3,1/3,1/2)$; $Fe_2Se_3O_{12}$: Z $(0,1/2,0)$, D $(0,1/2,1/2)$, B $(0,0,1/2)$, A $(-1/2,0,1/2)$, E $(-1/2,1/2,1/2)$; Fe_2BO_4: Z $(0,1/2,0)$, D $(0,1/2,1/2)$, B $(0,0,1/2)$, A $(-1/2,0,1/2)$, E $(-1/2,1/2,1/2)$; $FeSbO_4$: Z $(0,0,1/2)$, U $(1/2,0,1/2)$, X $(1/2,0,0)$, S $(1/2,1/2,0)$, R $(1/2,1/2,1/2)$, Y $(0,1/2,0)$, T $(0,1/2,1/2)$; MnP_2O_7: Z $(0,1/2,0)$, D $(0,1/2,1/2)$, B $(0,0,1/2)$, A $(-1/2,0,1/2)$, E $(-1/2,1/2,1/2)$; CrP_2O_6: C $(0.38,0.38,0)$, D $(-0.30,0.70,1/2)$, A $(0,0,1/2)$, M $(-1/2,1/2,1/2)$; $GaFeO_3$: X $(1/2,0,0)$, S $(1/2,1/2,0)$, R $(1/2,1/2,1/2)$, Z $(0,0,1/2)$, T $(0,1/2,1/2)$, Y $(0,1/2,0)$; $NiBiO_3$: X $(1/2,0,0)$, S $(1/2,1/2,0)$, R $(1/2,1/2,1/2)$, Z $(0,0,1/2)$, T $(0,1/2,1/2)$, Y $(0,1/2,0)$.

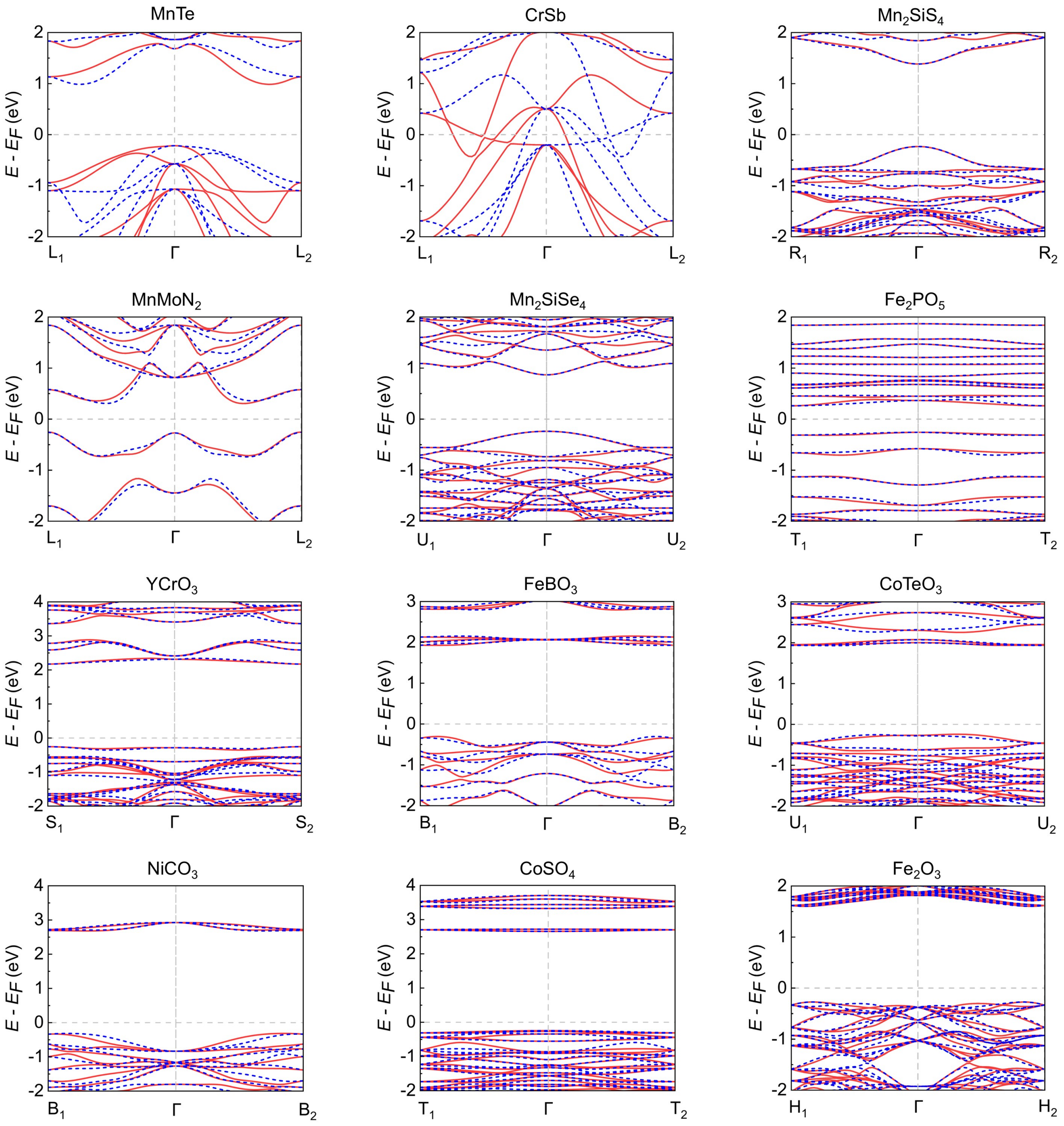

Figure S6. The electronic band structures of altermagnets MnTe, CrSb, Mn_2SiS_4, $MnMoN_2$, Mn_2SiSe_4, Fe_2PO5, $YCrO_3$, $FeBO_3$, $CoTeO_3$, $Nico_3$, $CoSO_4$, Fe_2O_3. The solid red and dashed blue lines represents the spin-up and spin-down channels, respectively.

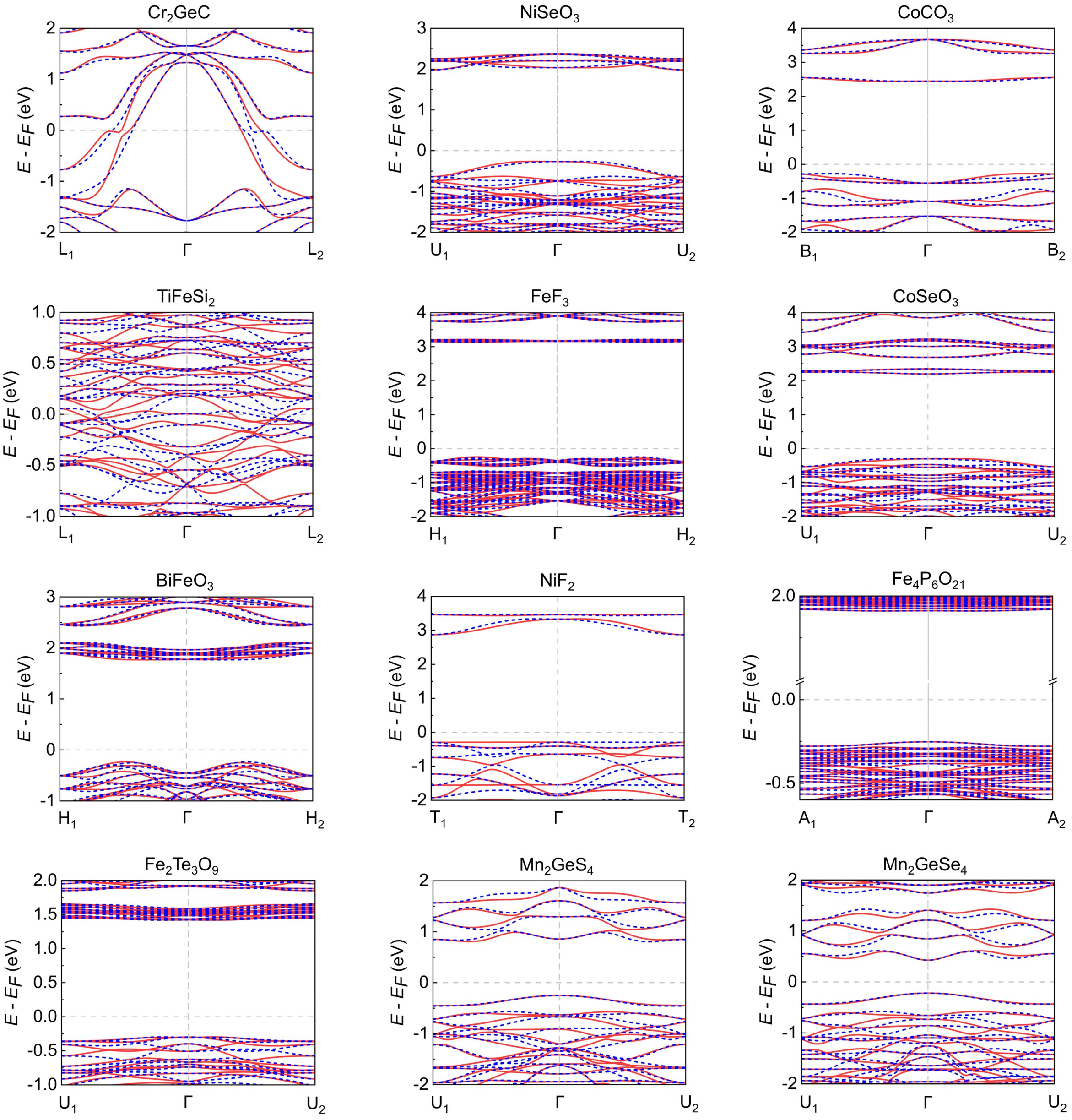

Figure S7. The electronic band structures of altermagnets Cr_2GeC, $NiSeO_3$, $CoCO_3$, $TiSeSi_2$, FeF_3, $CoSeO_3$, $BiFeO_3$, NiF_2, $Fe_4P_6O_21$, $Fe_2Te_3O_9$, Mn_2GeS_4, Mn_2GeSe_4. The solid red and dashed blue lines represents the spin-up and spin-down channels, respectively.

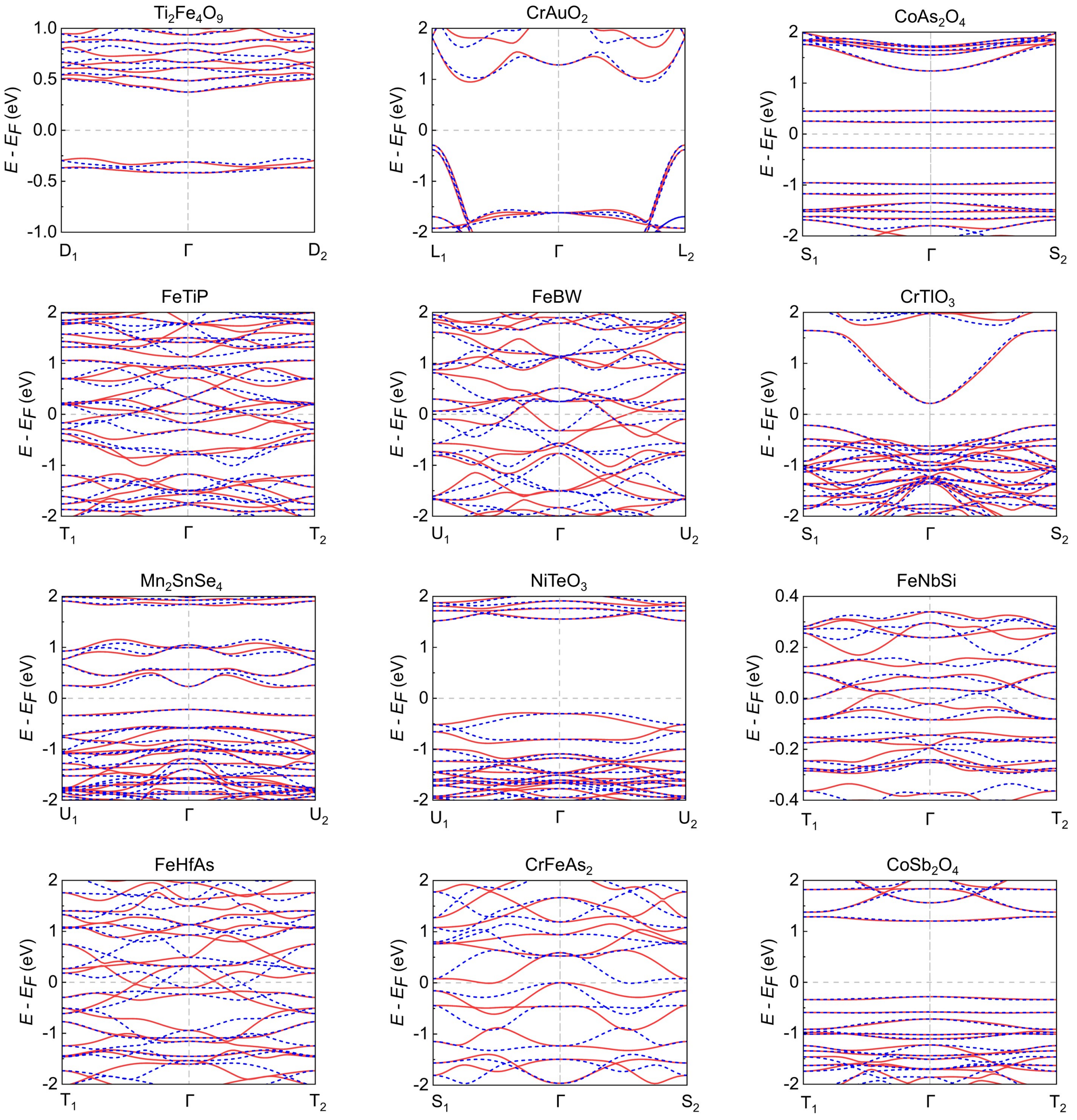

Figure S8. The electronic band structures of altermagnets $Ti_2Fe_4O_9$, $CrAuO_2$, $CoAs_2O_4$, FeTiP, FeBw, $CrTlO_3$, Mn_2SnSe_4, $NiTeO_3$, FeNbSi, HfFeAs, $CrFeAs_2$, $CoSb_2O_4$. The solid red and dashed blue lines represents the spin-up and spin-down channels, respectively.

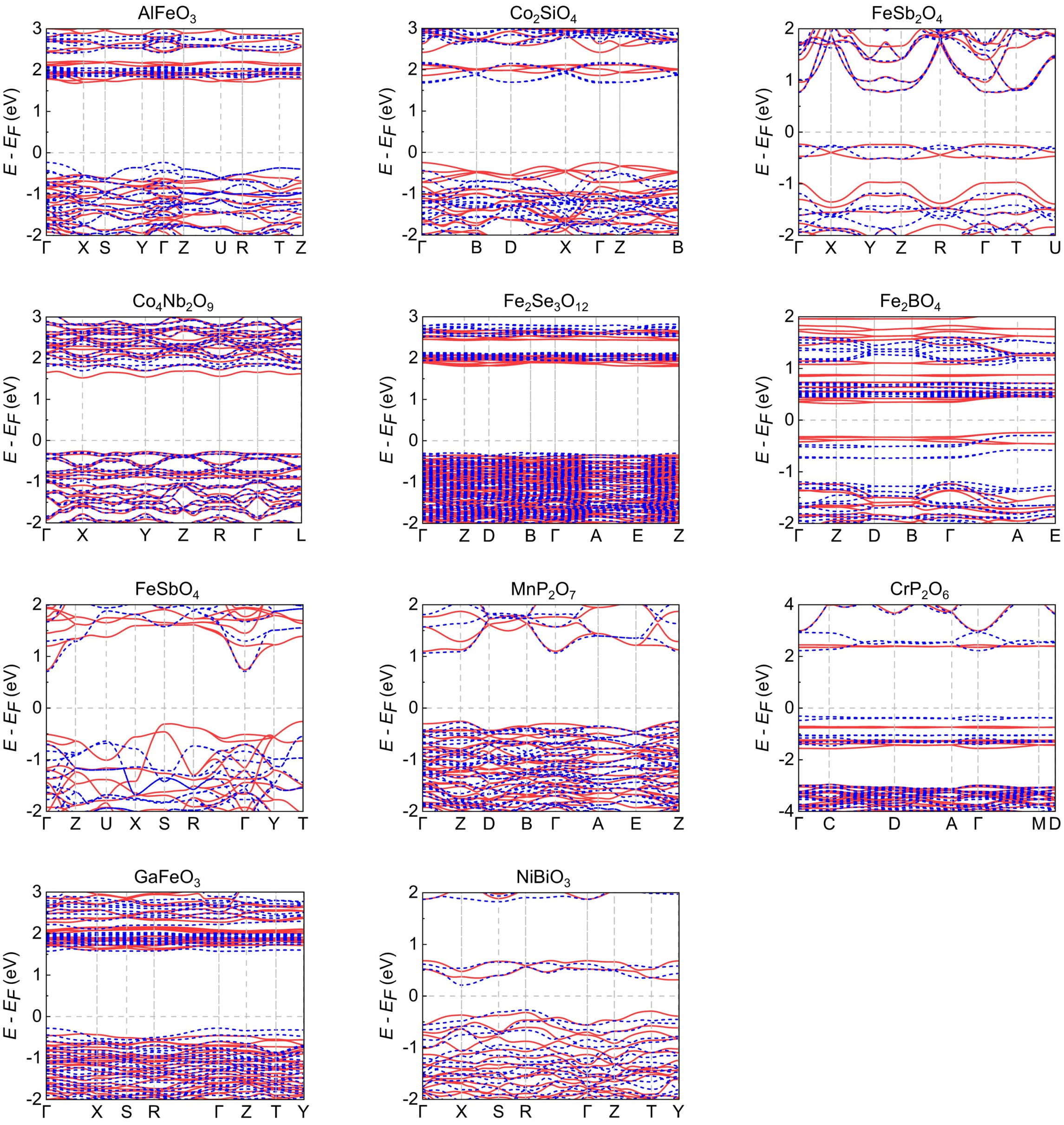

Figure S9. The electronic band structures of Luttinger-compensated ferrimagnets $AlFeO_3$, Co_2SiO_4, $FeSb_2O_4$, $Co_4Nb_2O_9$, $Fe_2Se_3O_1$2, Fe_2BO_4, $FeSbO_4$, MnP_2O_7, CrP_2O_6, $GaFeO_3$, $NiBiO_3$. The solid red and dashed blue lines represents the spin-up and spin-down channels, respectively.

Exchange couplings—Figure S10–S14 summarize the Heisenberg exchange J_{ij} as a function of the pair distance between magnetic atoms for all candidate compounds. Each curve corresponds to one representative magnetic site. The number of curves differs for distinct candidates because the number of magnetic sites with different chemical environments in the primitive cell is not identical. Compounds with only one exchange-distinct magnetic site are characterized by a single curve, whereas systems containing multiple chemically nonequivalent magnetic sites require multiple curves to describe their distinct J_{ij} distributions. Importantly, our calculations (Figure S10–S14) include all dominant exchange couplings, as evidenced by that the magnitude of J_{ij} decays toward zero with increasing pair distance in every case.

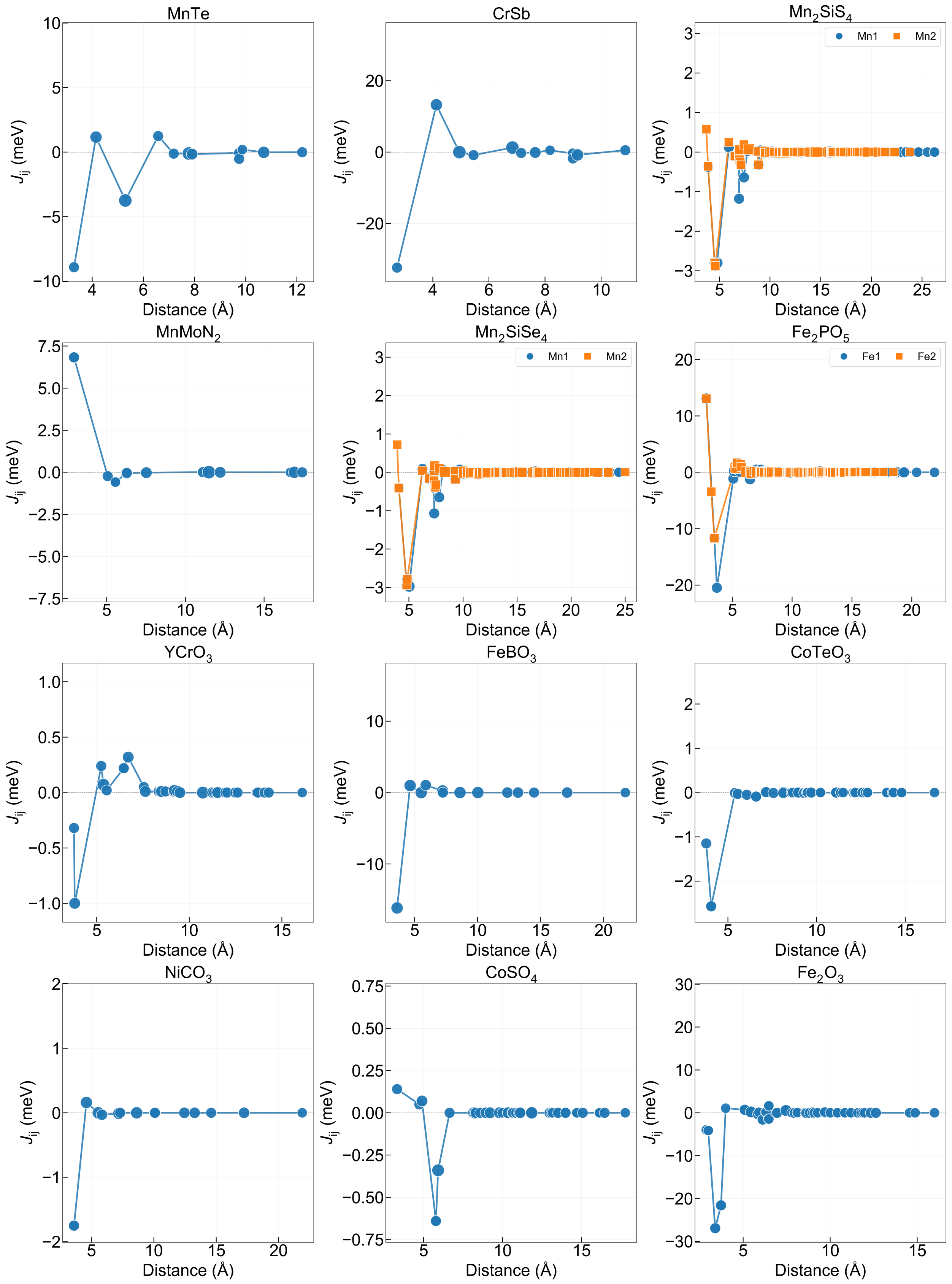

Figure S10. The exchange couplings of altermagnets MnTe, CrSb, Mn_2SiS_4, $MnMoN_2$, Mn_2SiSe_4, Fe_2PO5, $YCrO_3$, $FeBO_3$, $CoTeO_3$, $Nico_3$, $CoSO_4$, Fe_2O_3.

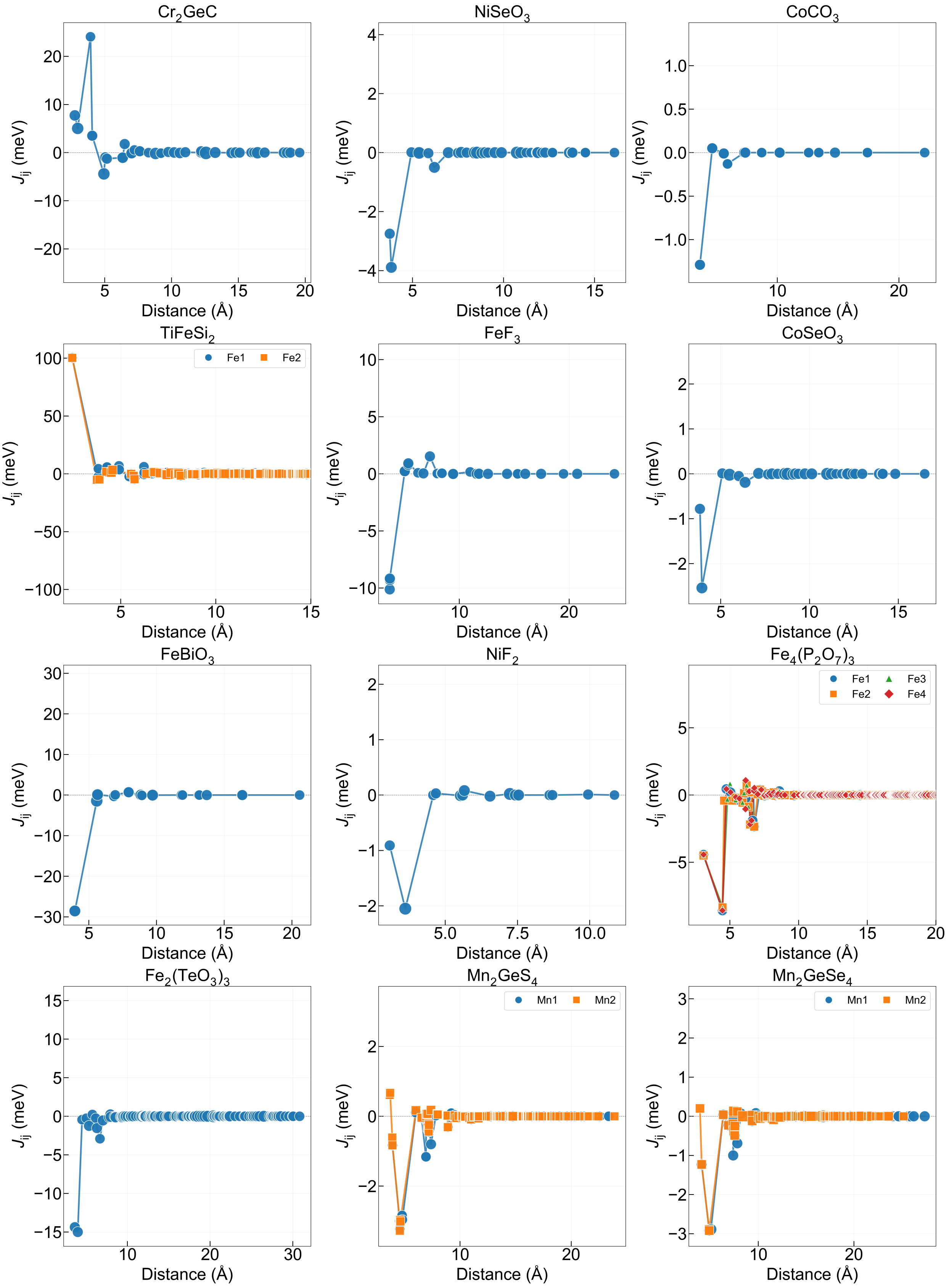

Figure S11. The exchange couplings of altermagnets Cr_2GeC, $NiSeO_3$, $CoCO_3$, $TiSeSi_2$, FeF_3, $CoSeO_3$, $BiFeO_3$, NiF_2, $Fe_4P_6O_2$1, $Fe_2Te_3O_9$, Mn_2GeS_4, Mn_2GeSe_4.

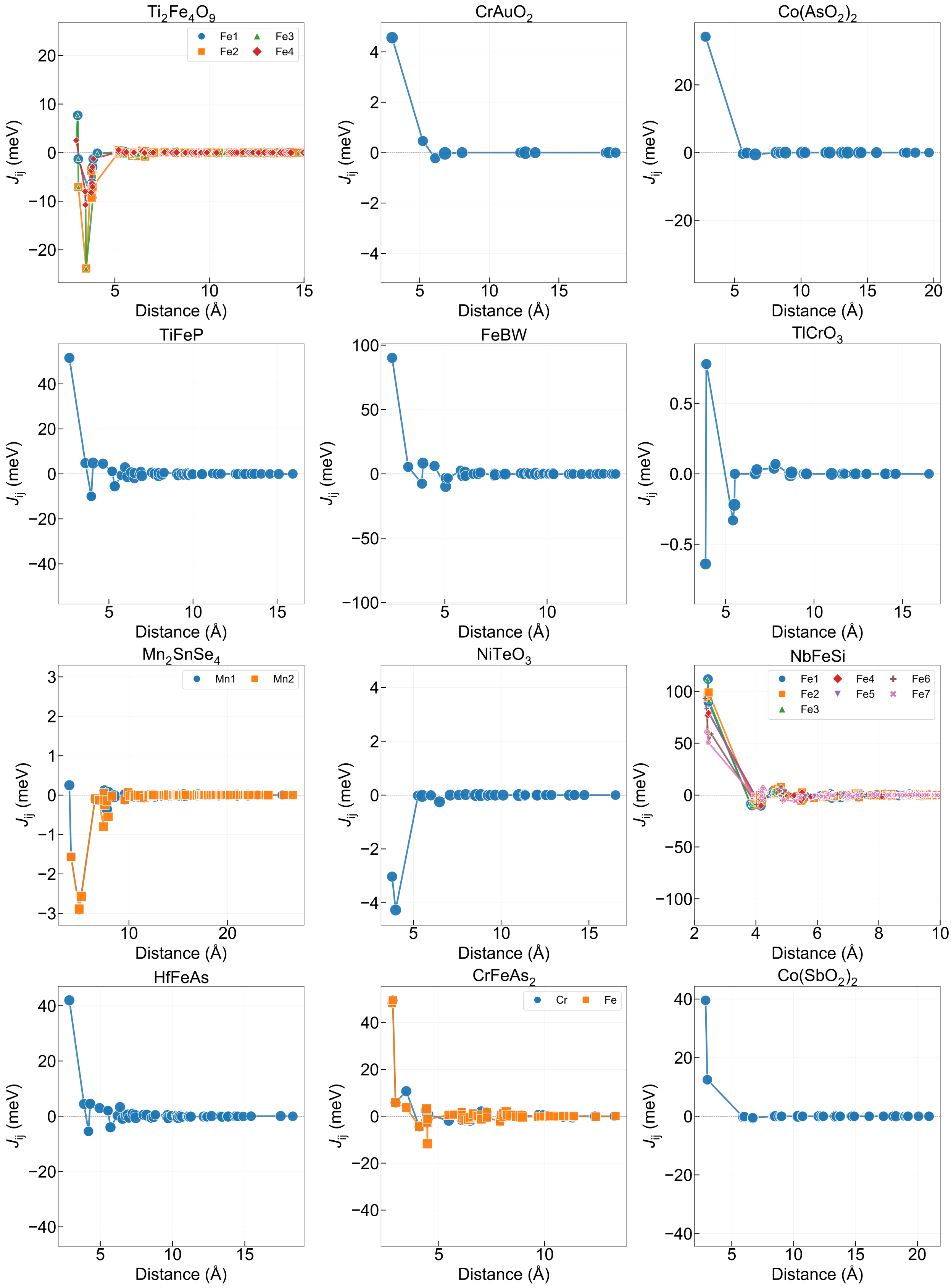

Figure S12. The exchange couplings of altermagnets Cr_2GeC, $NiSeO_3$, $CoCO_3$, $TiSeSi_2$, FeF_3, $CoSeO_3$, $BiFeO_3$, NiF_2, $Fe_4P_6O_2$1, $Fe_2Te_3O_9$, Mn_2GeS_4, Mn_2GeSe_4.

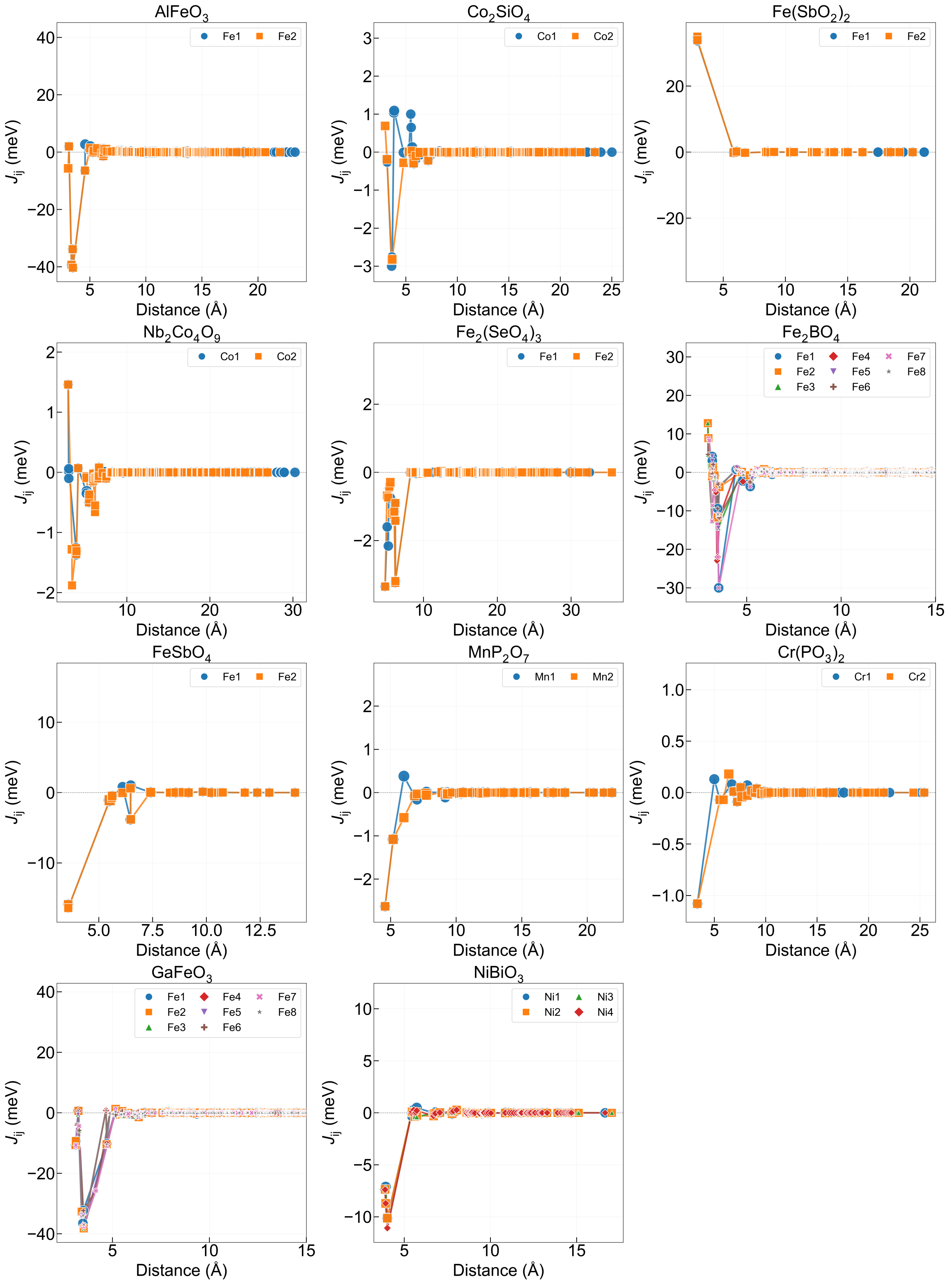

Figure S13. The exchange couplings of Luttinger-compensated ferrimagnets $AlFeO_3$, Co_2SiO_4, $FeSb_2O_4$, $Co_4Nb_2O_9$, $Fe_2Se_3O_1$2, Fe_2BO_4, $FeSbO_4$, MnP_2O_7, CrP_2O_6, $GaFeO_3$, $NiBiO_3$.

SOC effects in HfFeAs—Compared with the nonrelativistic calculations, SOC opens small gaps at several band-crossing points between the two opposite spin channels (Figure S14**a**), as expected for a compound containing relatively heavy elements. However, the overall band dispersion and the characteristic momentum-dependent spin splitting remain unchanged. In particular, the spin-split metallic bands near the Fermi level are still retained. Above features indicate that the underlying altermagnetic spin splitting is robust against SOC effects. We further recalculated the transverse spin Hall conductivity at the Fermi level. The value is $\sigma_{xy}^{s} = 446\ (\hbar/e)\,\Omega^{-1}\,\mathrm{cm}^{-1}$, which is close to the nonrelativistic value of $395\ (\hbar/e)\,\Omega^{-1}\,\mathrm{cm}^{-1}$. The limited difference indicates that SOC does not qualitatively affect the predicted spin Hall response. Therefore, HfFeAs remains a robust d-wave altermagnetic metal with sizable nonrelativistic spin Hall conductivity.

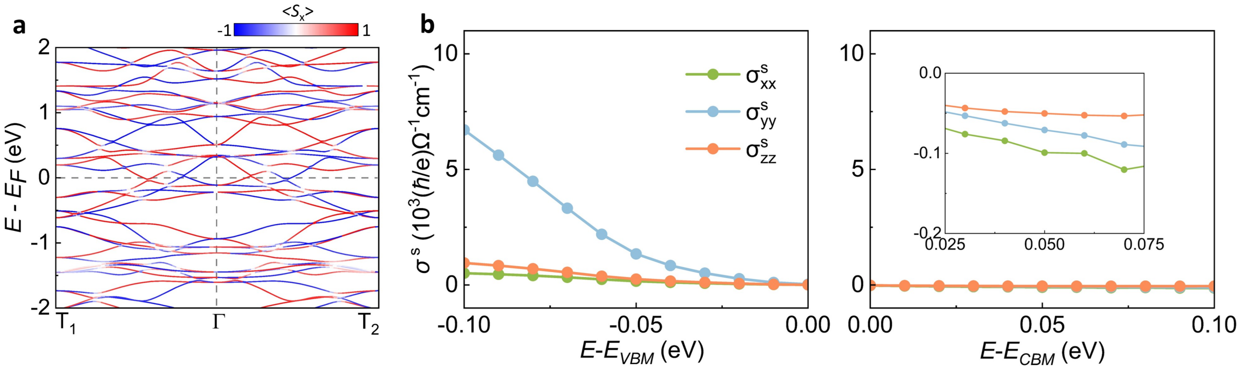

Figure S14. (a) The spin-resolved band structures of HfFeAs, where SOC effects are included in the DFT calculations. (b) The longitudinal spin conductivity of $FeSbO_4$ near the valence band maximum and conduction band minimum, respectively, indicating the doping-tunable spin transport.

The mean field estimation of critical temperatures—We estimate the Néel temperatures of HfFeAs and Co_2SiO_4 from the LKAG formula-resolved exchange couplings using the mean-field approximation. For magnetic sublattices α and β, the structure-dependent Fourier transform of the exchange couplings is defined as $J_{\alpha\beta}(\mathbf{q}) = \sum_{\mathbf{R}} J_{0\alpha,\mathbf{R}\beta} \exp\left[i\mathbf{q}\cdot(\mathbf{R}+\mathbf{r}_\beta-\mathbf{r}_\alpha)\right]$. The magnetic ordering wave vector is thus identified by the largest eigenvalue of $J(\mathbf{q})$ which minimizes the system energy. Near the magnetic transition, linearization of the classical mean-field equations gives $m_\alpha = \frac{1}{3k_\mathrm{B}T}\sum_\beta J_{\alpha\beta}(\mathbf{q}_N)m_\beta$. This equation has a nonzero magnetic solution when $T_N^{\mathrm{MFA}} = \frac{\lambda_{\max}[J(\mathbf{q}_N)]}{3k_\mathrm{B}}$, where $\lambda_{\max}[J(\mathbf{q}_N)]$ indicates the largest eigenvalue of the exchange matrix at the ordering wave vector. Applying this expression, we obtain $T_N \approx 452$ K for HfFeAs and $T_N \approx 63$ K for Co_2SiO_4.

Screening criteria—We define the DOS spin polarization as:

$$P(E) = \frac{D_\uparrow(E) - D_\downarrow(E)}{D_\uparrow(E) + D_\downarrow(E)} \times 100\%,$$

where $D_\uparrow(E)$ and $D_\downarrow(E)$ are the spin-up and spin-down DOS, respectively. The sign of $P(E)$ specifies the dominant spin channel, whereas $|P(E)|$ measures the magnitude of spin polarization. A point-like 100% polarization exactly at the VBM or CBM is not sufficient for the finite temperature carrier injection, because an infinitesimal spin splitting could produce apparently perfect spin selectivity over a vanishingly narrow energy interval. Instead, the spin polarization should remain high over a finite thermal window relevant for carrier occupation at the operating temperature [1]. Starting from the Fermi–Dirac distribution

$$f(E) = \left[\exp\left(\frac{E-\mu}{k_\mathrm{B}T}\right) + 1\right]^{-1},$$

the finite temperature occupation broadening is governed by its derivative,

$$-\frac{\partial f}{\partial E} = \frac{1}{4k_\mathrm{B}T}\,\mathrm{sech}^2\left(\frac{E-\mu}{2k_\mathrm{B}T}\right).$$

The half-maximum condition $sech^2(x) = 1/2$ gives approximately $3.53k_BT$. At 300 K, this corresponds to 0.091 eV, which rounds to the value of 0.10 eV adopted here as a physically motivated room-temperature screening window.

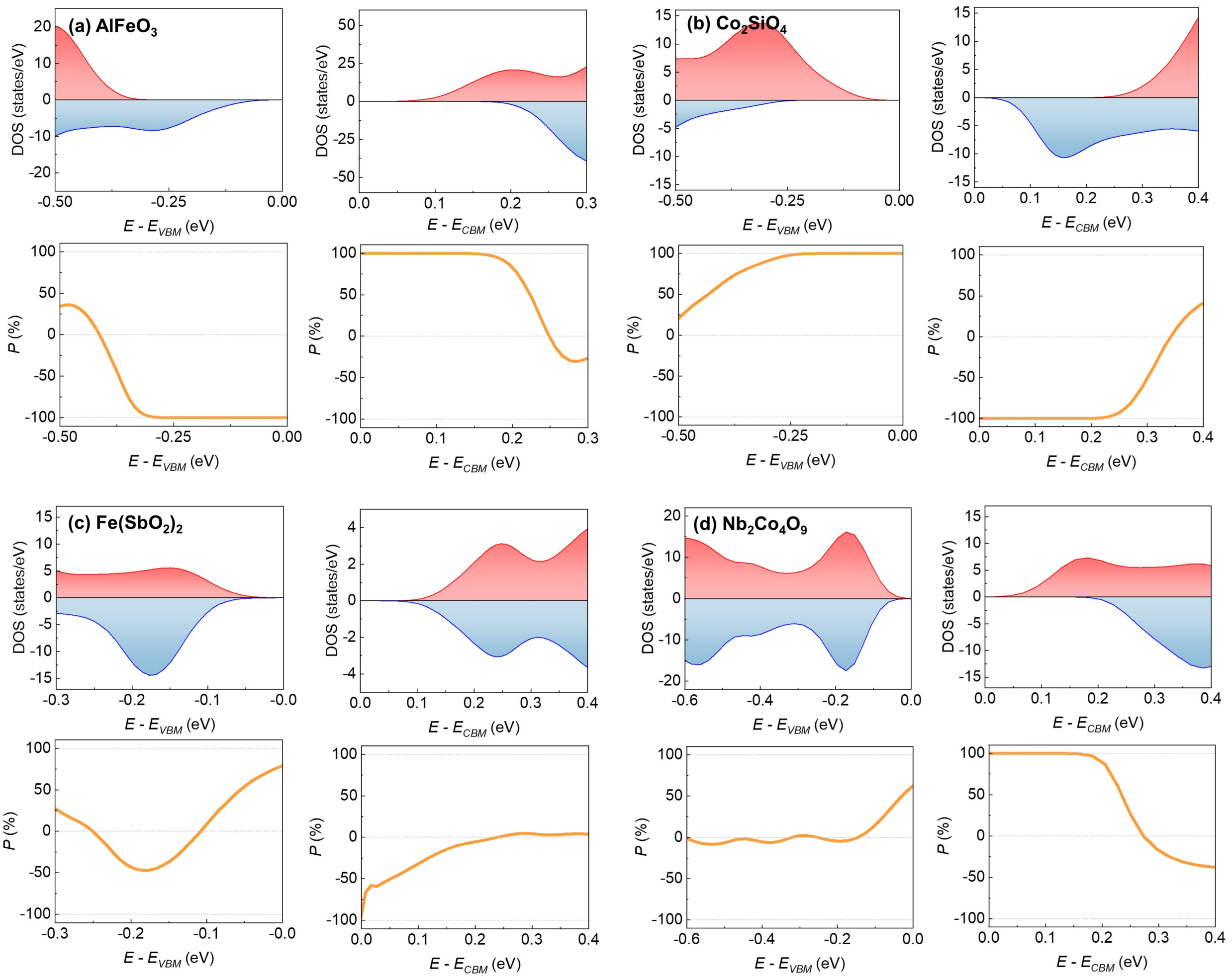

Figure S15. The spin-polarized density of states nearby the valence bands maximum and conduction bands minimum (up panel) and the corresponding spin polarization rate (down pannel) for (a) $AlFeO_3$, (b) Co_2SiO_4, (c) $Fe(SbO_2)_2$ and (d) $Nb_2Co_4O_9$.

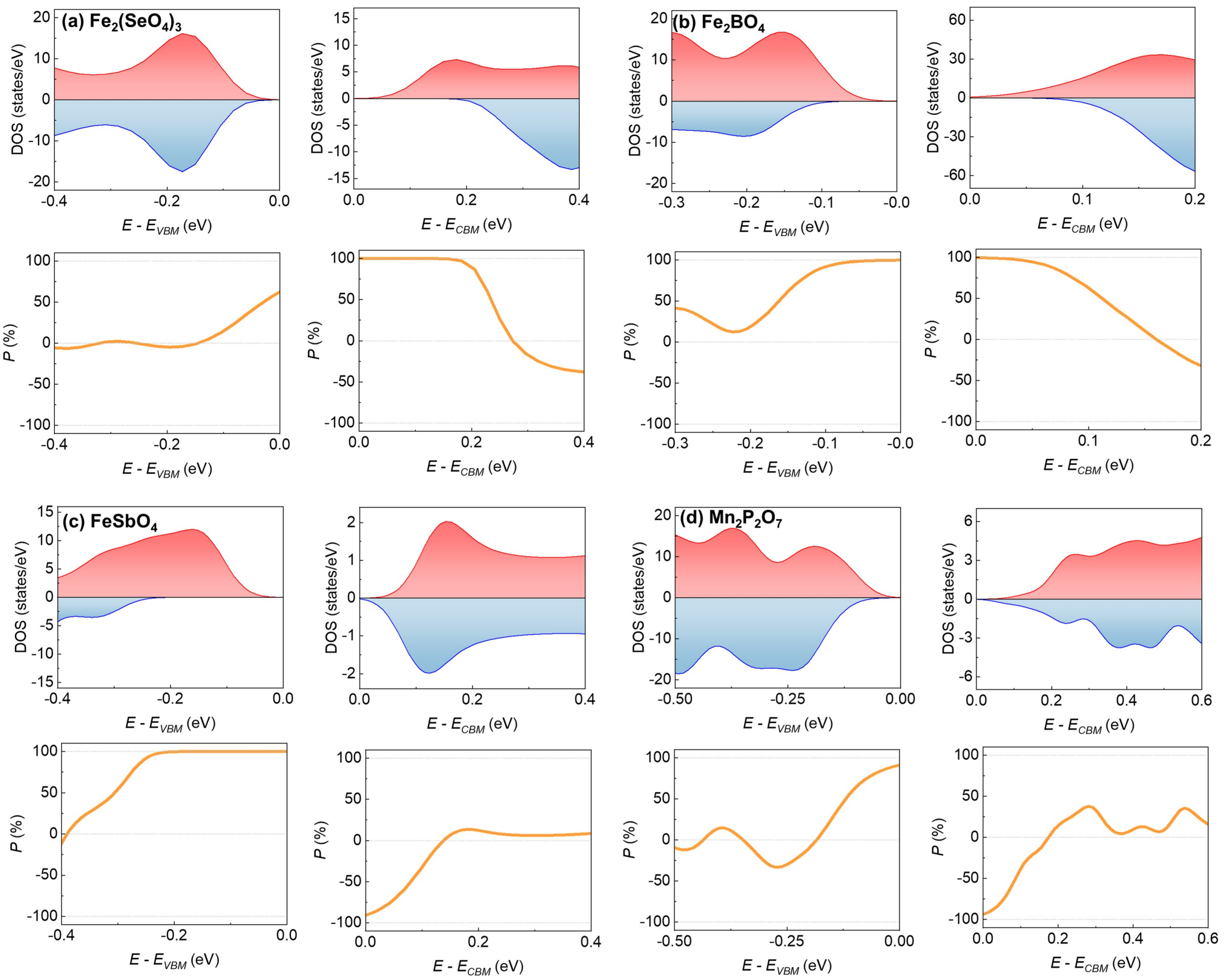

Figure S16. The spin-polarized density of states nearby the valence bands maximum and conduction bands minimum (up panel) and the corresponding spin polarization rate (down pannel) for (a) $Fe_2(SeO_4)_3$, (b) Fe_2BO_4, (c) $FeSbO_4$ and (d) $Mn_2P_2O_7$.

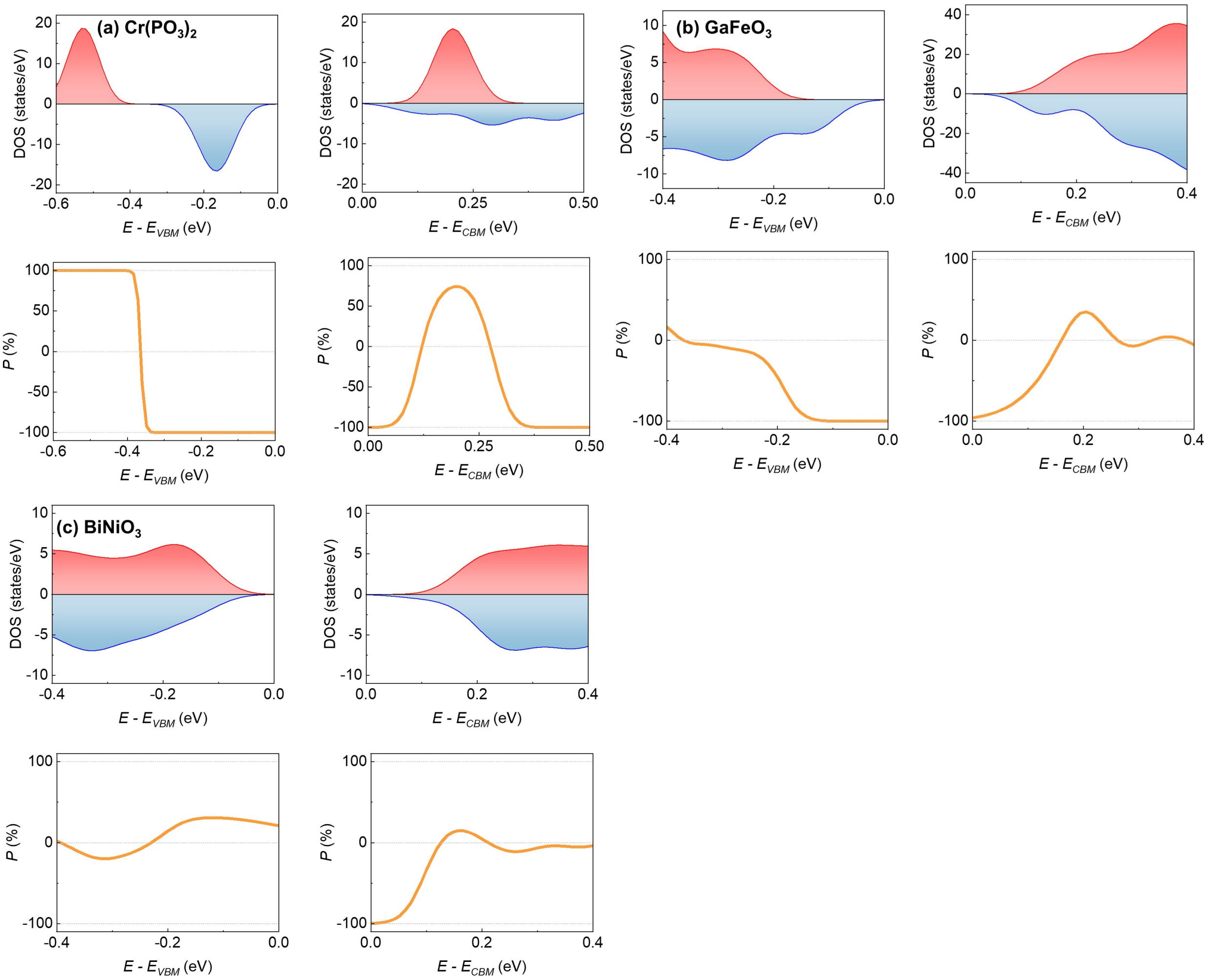

Figure S17. The spin-polarized density of states nearby the valence bands maximum and conduction bands minimum (up panel) and the corresponding spin polarization rate (down pannel) for (a) $Cr_2(PO_3)_2$, (b) $GaFeO_3$ and (c) $BiNiO_3$

Experimental Benchmark—We construct an experimental benchmark to substantiate the reliability of the ground-state determination method. The benchmark is assembled from multiple sources including MAGNDATA, the experimental benchmark of Horton et al. for magnetic ground-state prediction, benchmark data from the NovoMag Magnetic Materials Database, and well-known ferromagnetic Heusler alloys and intermetallic compounds [2–5]. A material is included only when the following conditions are satisfied: (i) the corresponding compound is present in our LKAG-completed set; (ii) the reported magnetic state is experimentally established or widely accepted in the literatures; (iii) the structure can be mapped unambiguously to the Materials Project entry used in our calculations; and (iv) the magnetic state can be assigned to the same class labels used in our workflow, namely FM, AFM, or noncollinear. After applying these criteria, the expanded benchmark contains 50 materials. The class distribution is 25 ferromagnets, 18 antiferromagnets, and 7 noncollinear magnets. Specifically, The 25 correctly matched ferromagnets are CrI_3, MnSb, CoS_2, Co_2FeSi, Co_2MnSi, Co_2MnSn, CrO_2, Fe_3Si, Fe_3Ge, Fe_3Pt, CoPt, YCo_5, MnAl, FeNi, YCo_3, $MnFe_4Si_3$, Mn_5Ge_3, Co_2CrSi, Co_2FeAl, Co_2MnAl, Co_2MnGa, Co_2FeGe, Ni_2MnGa, MnAs, and FePd. The 16 matched antiferromagnets are MnTe, CrSb, NiO, CoO, Fe_2O_3, $YCrO_3$, $MnPS_3$, CrN, $MnPd_2$, $TlCrO_3$, Fe_2PO_5, $FeBO_3$, $NiCO_3$, $CoCO_3$, FeF_3, and Mn_4N. The two antiferromagnets that are not correctly reproduced are CoF_2 and Cr_2O_3. Both are experimentally AFM but are predicted as FM. The 7 matched noncollinear materials are NbMnP, Pb_2MnO_4, $MnTe_2$, Mn_3As, $Co_2V_2O_7$, $CoGeO_3$, and $FePO_4$. The workflow correctly reproduces 48 out of these 50 experimental magnetic ground states, corresponding to an overall agreement of 96.0 %.

References

[1] N. W. Ashcroft and N. D. Mermin, *Solid State Physics* (Holt, Rinehart and Winston, New York, 1976).

[2] S. V. Gallego, J. M. Perez-Mato, L. Elcoro, E. S. Tasci, R. M. Hanson, K. Momma, M. I. Aroyo, and G. Madariaga, “MAGNDATA: Towards a Database of Magnetic Structures. I. The Commensurate Case,” *J. Appl. Cryst.* 49 (2016): 1750–1776.

[3] M. K. Horton, J. H. Montoya, M. Liu, and K. A. Persson, “High-Throughput Prediction of the Ground-State Collinear Magnetic Order of Inorganic Materials Using Density Functional Theory,” *npj Comput. Mater.* 5 (2019): 64.

[4] M. Sakurai, R. Wang, T. Liao, C. Zhang, H. Sun, Y. Sun, H. Wang, X. Zhao, S. Wang, B. Balasubramanian, X. Xu, D. J. Sellmyer, V. Antropov, J. Zhang, C.-Z. Wang, K.-M. Ho, and J. R. Chelikowsky, “Discovering Rare-Earth-Free Magnetic Materials through the Development of a Database,” *Phys. Rev. Mater.* 4 (2020): 114408.

[5] K. Elphick, W. Frost, M. Samiepour, T. Kubota, K. Takanashi, H. Sukegawa, S. Mitani, and A. Hirohata, “Heusler Alloys for Spintronic Devices: Review on Recent Development and Future Perspectives,” *Sci. Technol. Adv. Mater.* 22 (2021): 235–271.
